# Single Nucleotide Polymorphisms in the Wilms’ Tumour Gene 1 in Clear Cell Renal Cell Carcinoma

**DOI:** 10.1371/journal.pone.0058396

**Published:** 2013-03-06

**Authors:** Xingru Li, Sihan Wang, Raviprakash T. Sitaram, Charlotta Andersson, Börje Ljungberg, Aihong Li

**Affiliations:** 1 Department of Medical Biosciences, Clinical Chemistry, Umeå University, Umeå, Sweden; 2 Department of Perioperative Sciences, Urology and Andrology, Umeå University, Umeå, Sweden; 3 Department of Medical Biosciences, Pathology, Umeå University, Umeå, Sweden; AMS Biotechnology, United Kingdom

## Abstract

The Wilms’ tumour gene 1 (*WT1*) single nucleotide polymorphism (SNP) rs16754 has recently been described as an independent prognostic factor in acute myeloid leukaemia (AML) patients. It is of great interest to test whether *WT1* SNPs can be used as a molecular marker in other cancer types in order to improve risk and treatment stratification. We performed sequencing analysis on all 10 exons of the *WT1* gene in a total of 182 patients with clear cell renal cell carcinoma (ccRCC). Six different SNPs were identified, in descending order for minor allele frequency: rs2234582, rs16754, rs1799925, rs5030315, rs2234583, and rs2234581. At least one minor allele for *WT1* SNP was identified in 61% of ccRCC patients. In the entire study population, only 6% carried two copies of the minor allele. The genotypes of *WT1* SNPs in 78 tumour-free kidney tissue specimens were found to be in 95% concordance with corresponding tumour samples. No correlation was observed between *WT1* SNP genotypes and RNA expression level. *WT1* SNP genotypes did not associate with clinical and pathological characteristics. We found favourable outcomes associated with the homozygous minor allele for *WT1* SNP. However, SNP genotypes did not show to be of prognostic significance when comparing wild-type versus homozygous or heterozygous for the minor allele in the entire cohort. None of the previously reported *WT1* mutations in AML was found in the present study. A novel *WT1* missense mutation was identified in only one patient. Our data suggest that common *WT1* mutations are not involved in ccRCC. Due to too few cases harbouring the homozygous minor allele, the prognostic impact needs to be verified in larger study populations.

## Introduction

Renal cell carcinoma (RCC) represents about 3% of all adult malignancies [1]. The main subtypes of RCC are clear cell (75%), papillary (10%) and chromophobe (5%) [2]. In Sweden, patient survival has improved during the last decade and the 5-year relative survival rate for renal cancer is 55% for men and 58% for women [3].

Previous studies have demonstrated genetic abnormalities in clear cell renal cell carcinoma (ccRCC), of which inactivation of the tumour suppressor gene von Hippel-Lindau (VHL) plays a role in the pathogenesis [4]. Inactivation of VHL can occur through hypermethylation or mutations, including deletions, insertions, missense, nonsense and splice junction alterations [5]. VHL mutations were detected in 57% of ccRCC [6]. There was no significant association between mutation type and clinical characteristics [7].

The Wilms’ tumour gene 1 (*WT1*) is an important regulator of cell growth and development in the embryo kidney, adult urogenital system and central nervous system [8]. In 1990, *WT1* was first described as a tumour suppressor gene in Wilms’ tumour [9]. We have previously demonstrated that *WT1* can act as a tumour suppressor in RCC via multiple pathways leading to down-regulation of *hTERT* [10]. However, subsequent research has revealed that *WT1* may function as an oncogene in other types of cancers including leukaemia and breast cancer [11]. Thus, *WT1* was recently proposed to act as a chameleon gene in malignancies [12].

The *WT1* gene is located on chromosome 11p13, contains 10 exons and encodes a 49–52 kDa protein. Sequencing analysis demonstrated that *WT1* mutations were shown in only 10% of sporadic Wilms’ tumours [13]. However, *WT1* mutations are frequently found in certain urogenital anomaly syndromes such as Denys-Drash syndrome [14] and Frasier syndrome [15]. *WT1* mutations have also been demonstrated in approximately 10% of T-acute lymphoblastic leukaemia (T-ALL) [16]–[17] and acute myeloid leukaemia (AML) patients [18]. Furthermore, AML patients with mutations in *WT1* were significantly associated with worse relapse-free survival and overall survival (OS) [19]–[22]. Recently, elevated clinical interests in leukaemia have been shown regarding the prognostic impact of a single nucleotide polymorphism (SNP) rs16754 in *WT1* exon 7. In a German study, cytogenetically normal AML patients with rs16754 (AG) and rs16754 (AA) genotypes were found to have better outcome compared to patients with rs16754 (GG) genotype [23]. In a large Cancer and Leukemia Group study, AML patients with rs16754 (GG) genotype had a more favourable outcome in a subset of patients with *FLT3-ITD* [24]. However, in a Korean cohort, the different genotypes of rs16754 did not have any significant impact on clinical outcome in AML [25].

To test whether *WT1* SNP genotypes are also associated with outcome in ccRCC, we investigated the role of *WT1* SNPs as candidate polymorphisms for survival in 182 patients in the context of other clinical parameters. Six different SNPs in *WT1* were identified and we demonstrated at least one or two copies of the minor allele in 61% of ccRCC tumour samples. *WT1* SNP genotypes did not correlate to clinical and pathological characteristics and no differences were demonstrated between patients with wild-type versus homozygous or heterozygous for the minor allele in relation to OS and disease-specific survival (DSS) in the entire cohort. In addition, we observed favourable outcome associated with homozygous minor allele.

## Materials and Methods

### Ethics Statement

This study was approved by the Human Ethics Committee of the Medical Faculty, Umeå University, Sweden (2007-071M).

### Patients and Tissue Samples

The study included 182 adult patients who were diagnosed with ccRCC between 1985 and 2007. These patients were treated at Umeå University Hospital, Umeå, Sweden based on guidelines from the European Association of Urology [26]. The median age of the patients was 65.5 years (range 38–87 years) and median survival time was 49.5 months (range 1–300 months). For patients providing corresponding tumour-free specimens the median age was 67 years (range 38–87 years) and median survival time of 55.5 months (range 1–115 months).

A total of 260 tissue specimens including 182 ccRCC tumour samples and 78 corresponding tumour-free renal cortical tissue samples were sequenced for *WT1* exons. The histological grading of specimens was performed according to the Fuhrman system [27]. Tumour stages were classified according to the TNM classification 2002 [28]. Follow-up medical records of the patients were used for survival analysis.

### DNA and RNA Preparation

According to the manufacturer’s instructions, DNA was extracted from frozen tissue specimens using MagAttract DNA Mini M48 Kit with Qiagen BioRobot M48 (QIAGEN, Hilden, Germany). Total RNA was isolated using the TRIzol method (Invitrogen, Stockholm, Sweden). After extraction and isolation, the DNA and RNA were stored at −80°C until use. cDNA was synthesized by reverse transcription with the Superscript II Reverse Transcriptase kit according to the manufacturer’s protocol (Invitrogen).

### Sequencing Analysis of the *WT1* Genes

Using intron-exon flanking primer pairs, polymerase chain reaction (PCR) technique was applied to amplify the whole coding region of 10 exons of the *WT1* gene. Twelve pairs of primers were previously described [29]. Because of GC-rich sequences of exon 1, Hot Star Plus polymerase (QIAGEN, Hilden, Germany) was used for DNA amplification as previously described [29]; for the remaining exons 2 to 10, AmpliTaq Gold polymerase (Applied Biosystems, Foster City, CA, USA) was used. The total reaction volume of 25 µL contained approximately 50 ng DNA, 10 pmol of each primer, deoxynucleoside triphosphates (10 mM each), and Hot Star Plus polymerase (QIAGEN) or AmpliTaq Gold polymerase (Applied Biosystems) with supplied buffers. DNA was amplified using the following PCR conditions: 95°C for 10 minutes, 40 cycles of 95°C for 45 seconds, 57°C for 30 seconds, and 72°C for 45 seconds; and finally 72°C for 7 minutes. PCR products were purified by standard methods and subsequently sequenced using Big Dye Terminator v3.1 Cycle Sequencing Kit (Applied Biosystems) with M13 forward and reverse primer. Sequence reactions were analyzed on an Applied Biosystems 3730xl DNA Analyzer (Applied Biosystems). Nucleotide sequences were aligned using the Sequencher software v4.7 (Gene Codes Corporation, Ann Arbor, MI, USA). The derived *WT1* gene sequences were identified by comparison with the corresponding reference genes using GenBank (EMBL) (http://www.ncbi.nlm.nih.gov/genbank/), BLAST (http://blast.ncbi.nlm.nih.gov/ *)* and dbSNP (http://www.ncbi.nlm.nih.gov/snp) searches.

### *WT1* RNA Expression Using Real-time Quantitative PCR (RQ-PCR)

Quantitative analysis of *WT1* RNA expression was performed using TaqMan technology in the 7900 HT Real-Time PCR System (Applied Biosystems, Foster City, CA, USA). RQ-PCR reactions were carried out in a 25 µL volume containing 12.5 µL universal PCR master mix, each primer at a concentration of 0.5 µM, probe at 0.1 µM, and 50 ng of cDNA. Amplification conditions, primers and probes for the *WT1* gene were previously described [10], [30]. Standard curves were generated using a series of dilutions of plasmid DNA carrying the *WT1* or *β-actin* gene with copy numbers from 10^0^ to 10^7^. The mean of triplicates of the *WT1* gene copy numbers was divided by the mean of duplicates of copy numbers of the *β-actin* gene.

### Statistical Analysis

Statistical analysis was performed using SPSS (version 18) statistical software. The Kruskal-Wallis test was used to compare differences in the expression of independent variables. The χ^2^ test was used to test the significance of observed differences in proportions. The Kaplan-Meier method was used to estimate the distribution of DSS and OS. Differences in survival distribution between different *WT1* SNP genotype groups were compared using the log-rank test. OS was defined as being from the date of diagnosis to death of any cause, and DSS was defined as being from the date of diagnosis to death by disease.

## Results

### 1. Frequencies and Features of *WT1* SNPs in ccRCC Patients

Sequence analysis was performed on all 10 exons in *WT1* in 182 ccRCC tumour specimens. A total of six different *WT1* gene SNPs were identified (Table 1). The genotypes of each SNP met Hardy-Weinberg equilibrium (Table 1). One or two copies of the minor allele were found in 95 tumour specimens in exon 1, 52 in exon 7 and 27 in exon 10 (Table 1). The minor allele frequencies of these SNPs presented in order with 16.8% of rs2234582 in exon 1, followed by 16% of rs16754 in exon 7, 13.7% of rs1799925 in exon 1, 7.7% of rs5030315 in exon 10, 6.6% of rs2234583 in exon 1 and 0.6% of rs2234581 in exon 1 (Table 1). Similar frequencies of these SNPs were presented in 78 corresponding tumour-free renal cortical tissue samples except rs2234581 (Table 1). The majority of the minor allele of these SNPs was heterozygous in both tumour and tumour-free tissues (Table 1). At least one copy of the minor allele could be detected in 111 of the 182 (61%) tumour specimens.

**Table 1:** Characteristics of Wilms’ tumour gene 1 (*WT1*) single nucleotide polymorphism (SNP) in ccRCC tumour and corresponding tumour-free renal cortical tissues.

SNP	Exon	n	Minor allele frequency (%)		Heterozygous (%)		Homozygous (%)		Wild-type (%)		p value for HWE*
			Tumour	Tumour-free	Tumour	Tumour-free	Tumour	Tumour-free	Tumour	Tumour-free	
**rs2234581(C→A)**	1	2	**0.6%**	**–**	**1.1%**	**–**	**0%**	**–**	**98.9%**	**–**	**0.96**
**rs2234582(G→T)**	1	57	**16.8%**	**16.1%**	**29.1%**	**29.5%**	**2.2%**	**1.3%**	**68.7%**	**69.2%**	**0.66**
**rs1799925(C→T)**	1	45	**13.7%**	**12.9%**	**22%**	**23.1%**	**2.7%**	**1.3%**	**75.3%**	**75.6%**	**0.49**
**rs2234583(C→T)**	1	19	**6.6%**	**5.2%**	**12.1%**	**7.7%**	**0.5%**	**1.3%**	**87.4%**	**91%**	**0.91**
**rs16754(A→G)**	7	52	**16%**	**13.5%**	**25.3%**	**21.8%**	**3.3%**	**2.6%**	**71.4%**	**75.6%**	**0.57**
**rs5030315(A→G)**	10	27	**7.7%**	**5.8%**	**15.4%**	**11.5%**	**0**	**0**	**84.6%**	**88.5%**	**0.40**

SNP, single nucleotide polymorphism; HWE, Hardy-Weinberg equilibrium.

Furthermore, SNP genotypes in 78 tumour and corresponding tumour-free specimen pairs were compared. A high concordance (95%) was demonstrated (Table 2). One patient carried no rare SNPs in the tumour specimen but one heterozygous SNP minor allele G→T in rs2234582 in the corresponding tumour-free specimen. Another patient carried the minor allele of three SNPs in the tumour specimen and these three together with a fourth heterozygous genotype SNP rs16754 were detected in the corresponding tumour-free specimen. Two patients carried total of three or four minor alleles of the SNPs but only one SNP genotype differed in their tumour and tumour-free samples (Table 2).

**Table 2:** Comparable number of *WT1* SNP genotypes in 78 ccRCC tumour and corresponding tumour-free renal cortical tissue pairs.

Number of SNP*	Tumour specimens (%)	Corresponding tumour-free specimens (%)
0	36 (46.2%)	35 (44.9%)
1	13 (16.7%)	14 (17.9%)
2	25 (32%)	25 (32.1%)
3	3 (3.8%)	2 (2.6%)
4	1 (1.3%)	2 (2.6%)

SNP*, single nucleotide polymorphism with one or two copies of the minor allele.

### 2. No Correlation between *WT1* RNA Expression and SNP Genotypes

*WT1* RNA expressions were analyzed by RQ-PCR in a total of 115 tissue samples including 100 tumour and 15 tumour-free specimens. Similar to our previous findings [10], significantly lower RNA expression in ccRCC was observed in comparison with tumour-free renal cortical tissue (p = 0.001). *WT1* RNA expression was compared between different *WT*1 SNP genotype groups. No difference in *WT1* RNA expression levels was found in tumours (p = 0.726) and tumour-free tissue samples (p = 0.779, Table 3).

**Table 3:** No correlation between *WT1* SNP genotypes and *WT1* RNA expression in ccRCC.

Specimens	SNP genotypes		*WT1*/*β-actin* RNA level (×10^−3^)		p
		N	Median (range)	Mean ± SD	
**Tumours**					0.726
	Homozygous minor allele	7	0.057 (0.01–0.5)	0.129±0.18	
	Heterozygous minor allele	57	0.029 (0–2.52)	0.219±0.502	
	Wild-type	36	0.032 (0.001–1.316)	0.106±0.236	
**Tumour-free tissues**					0.779
	Homozygous minor allele	0	−	−	
	Heterozygous minor allele	7	1.245 (0.599–4.29)	1.758±1.252	
	Wild-type	8	1.335 (0.192–5.132)	1.547±1.561	

*WT1*, Wilms’ tumour gene 1; −, homozygous minor allele was not identified in tumour-free tissues.

### 3. No Correlation between *WT1* SNP Genotypes and Clinical and Pathological Characteristics in ccRCC Patients

*WT1* SNP genotypes and its relation to clinical and pathological characteristics are shown in Table 4. No significant differences were found between patients with wild-type and homozygous or heterozygous for the minor allele of the *WT1* SNPs with regard to age (p = 0.397), sex (p = 0.542), tumour stage (p = 0.947), tumour size (p = 0.602), tumour grade (p = 0.718), DSS (p = 1) and OS (p = 0.873).

**Table 4:** No correlation between *WT1* SNP genotypes and clinical and pathologic characteristics in patients with ccRCC.

	All patients	Homozygous	Heterozygous	Wild-type	p*
**n**	182	11 (6%)	100 (54.9%)	71 (39%)	
**Age** **(years)**					0.397
25–49	19(10.4%)	1 (0.5%)	13 (7.1%)	5 (2.7%)	
50–59	41(22.5%)	7 (3.8%)	20 (11%)	14 (7.7%)	
60–69	47(25.8%)	3 (1.6%)	26 (14.3%)	18 (9.9%)	
70–85	75(41.2%)	0	41 (22.5%)	34 (18.7%)	
**Sex**					0.542
Male	102(56%)	6 (3.3%)	54 (29.7%)	42 (23.1%)	
Female	80(44%)	5 (2.7%)	46 (25.3%)	29 (15.9%)	
**Tumour** **stage**					0.947
T1	58(31.9%)	5 (2.7%)	29 (15.9%)	24 (13.2%)	
T2	28(15.4%)	3 (1.6%)	14 (7.7%)	11 (6%)	
T3	43(23.6%)	1 (0.5%)	25 (13.7%)	17 (9.3%)	
T4	53(29.1%)	2 (1.1%)	32 (17.6%)	19 (10.4%)	
**Tumour** **size**					0.602
≥70 mm	117(64.3%)	9 (4.9%)	64 (35.2%)	44 (24.2%)	
<70 mm	65(35.7%)	2 (1.1%)	36 (19.8%)	27 (14.8%)	
**Tumour** **grade**					0.718
I	13(7.1%)	1 (0.5%)	7 (3.8%)	5 (2.7%)	
II	44(24.2%)	3 (1.6%)	21 (11.5%)	20 (11%)	
III	83(45.6%)	4 (2.2%)	47 (25.8%)	32 (17.6%)	
IV	42(23.1%)	3 (1.6%)	25 (13.7%)	14 (7.7%)	
**DSS**					1
Alive	60(40%)	8 (5.3%)	28 (18.7%)	24 (16%)	
Dead	90(60%)	2 (1.3%)	53 (35.3%)	35 (23.3%)	
**OS**					0.873
Alive	60(33%)	8 (4.4%)	28 (15.4%)	24 (13.2%)	
Dead	122(67%)	3 (1.6%)	72 (39.6%)	47 (25.8%)	

DSS, disease specific survival; OS, overall survival;

p*, significance compared between patients with *WT1* SNP homozygous or heterozygous for the minor allele versus wild-type.

### 4. Homozygous Minor Allele for *WT1* SNPs were Associated with Favourable Clinical Outcome

We investigated the prognostic impact of the *WT1* SNPs, in our whole cohort of 182 ccRCC patients, with a median OS of 49.5 months (range 1–300 months) and a median DSS of 49 months (range 1–293 months). In the subgroup of patients with homozygous minor allele (6%), median OS was 91 months (range 9–243 months) and median DSS was 96.5 months (range 9–243 months). Patients with heterozygous minor allele (54.9%) had a median OS of 41 months (range 1–300 months) and a median DSS of 40 months (range 1–276 months). Wild-type patients (39%) had a median OS of 49 months (range 1–293 months) and a median DSS of 48 months (range 2–293 months). We observed no significant differences in outcome between patients with wild-type versus homozygous or heterozygous for the minor allele (Table 4).

**Figure 1:**
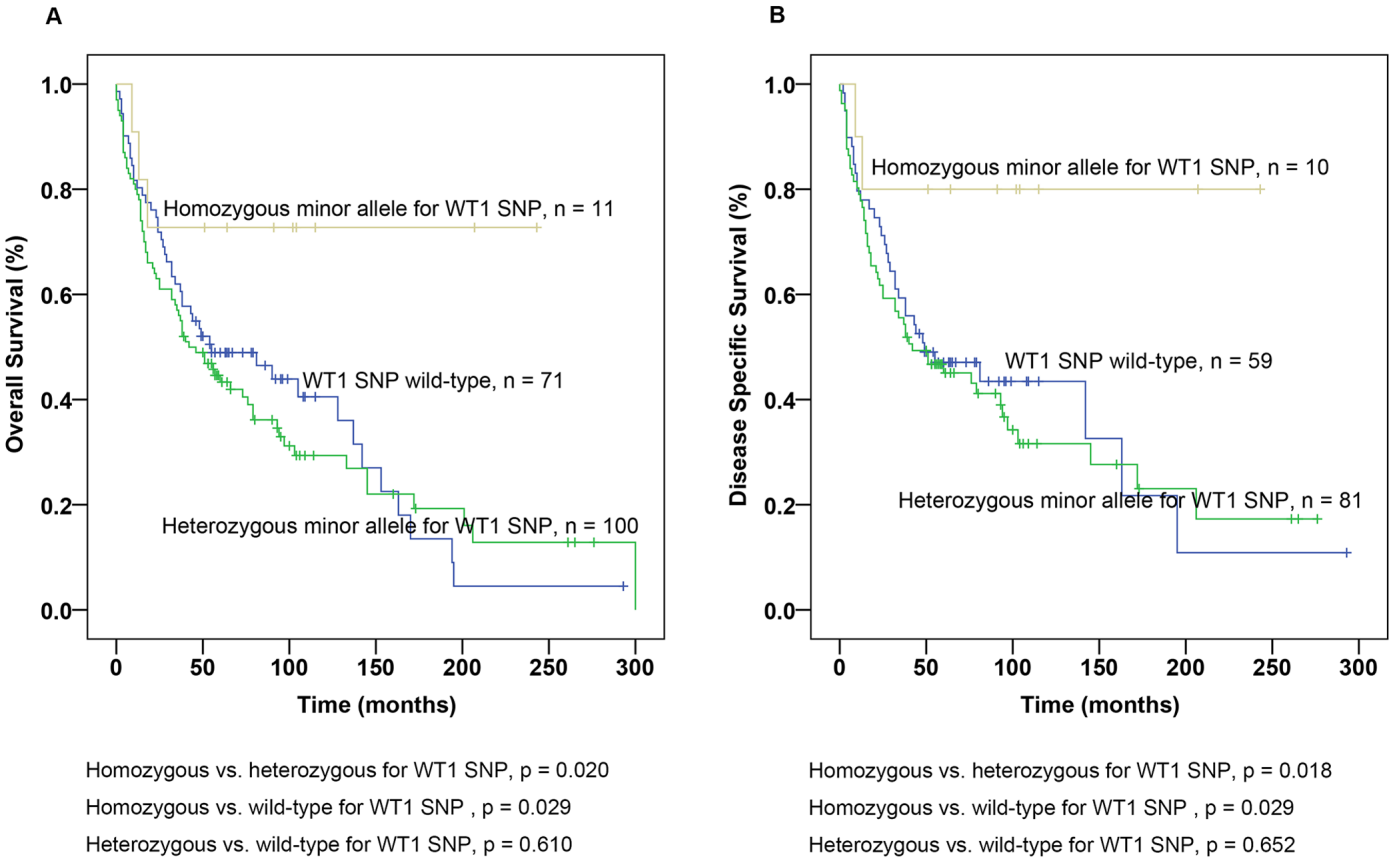
Survival analysis of Wilms’ tumour gene 1 (*WT1*) single nucleotide polymorphism (SNP) in ccRCC based on genotypes. (A) Kaplan-Meier curves of Overall Survival (OS) for patients with SNP genotypes in *WT1* and (B) Disease-specific survival (DSS) for patients with *WT1* SNP genotype.

**Figure 2:**
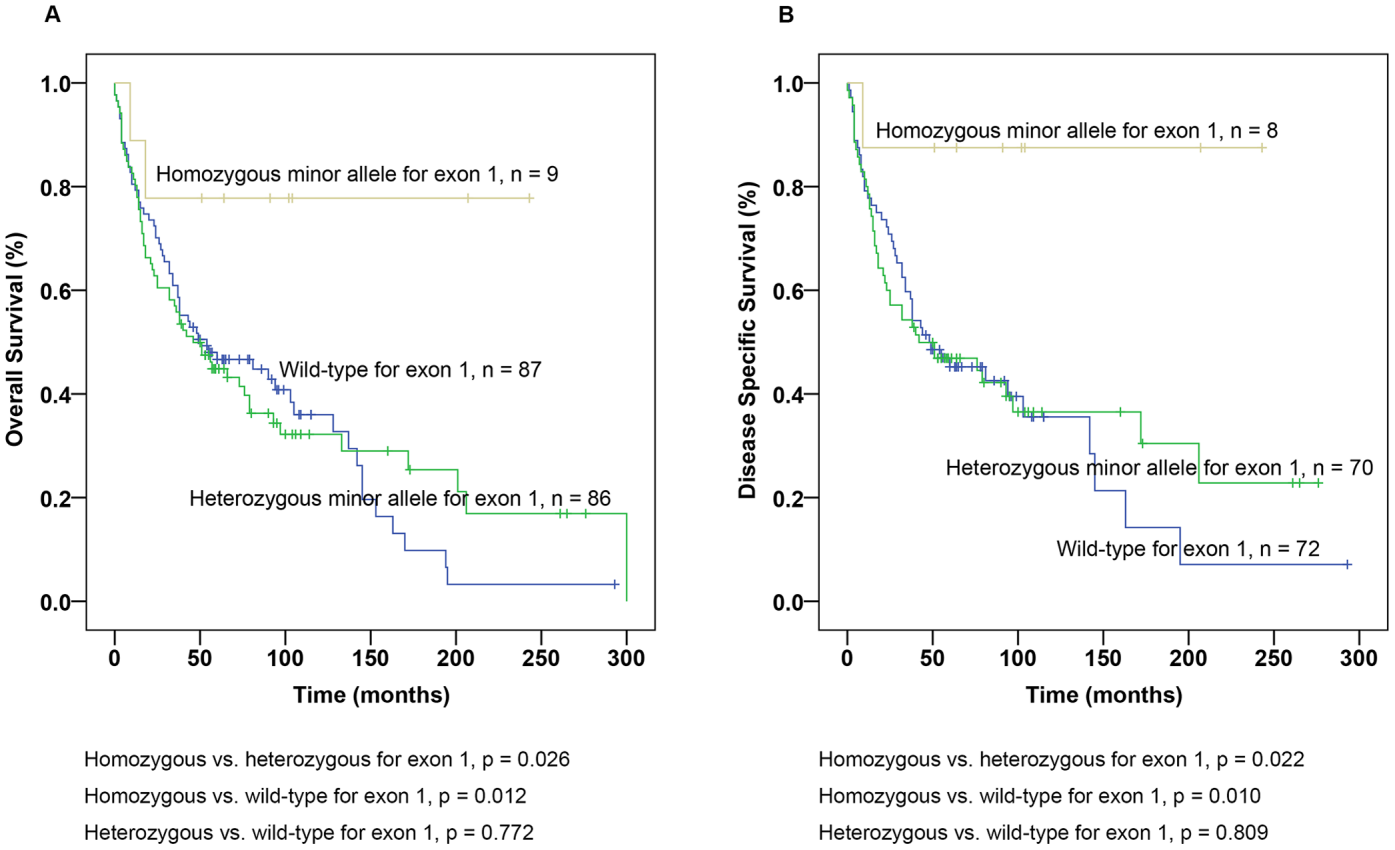
Prognostic impact of Wilms’ tumour gene 1 (*WT1*) single nucleotide polymorphisms (SNPs) in exon 1 in ccRCC. (A) Overall Survival (OS) for patients with wild-type, heterozygous and homozygous minor allele in exon 1 and (B) Disease-specific survival (DSS) for patients with wild-type, heterozygous and homozygous minor allele in exon 1.

Furthermore, we analysed the outcome associated with genotypes separately in *WT1* wild-type, heterozygous and homozygous for the minor allele. Patients with homozygous minor allele had longer OS and DSS than patients with heterozygous for the minor allele (p = 0.020 for OS and 0.018 for DSS, Figure 1A and B) and wild-type (p = 0.029 for OS and DSS, Figure 1A and B). No difference in OS or DSS was found between patients with *WT1* wild-type and heterozygous for minor allele (p = 0.610 for OS and p = 0.652 for DSS, Figure 1A and B). In addition, we combined patients with different *WT1* genotypes in exon 1 and performed Kaplan-Meier analysis. Patients with homozygous allele for *WT1* in exon 1 were observed to have a favourable outcome for both OS and DSS compared to patients with heterozygous (p = 0.026 for OS and p = 0.022 for DSS, Figure 2A and B) and wild-type (p = 0.012 for OS and p = 0.010 for DSS, Figure 2A and B). Patients with wild-type and heterozygous genotypes in exon 1 did not differ significantly in OS or DSS (p = 0.772 for OS and p = 0.809 for DSS, Figure 2A and B).

**Figure 3:**
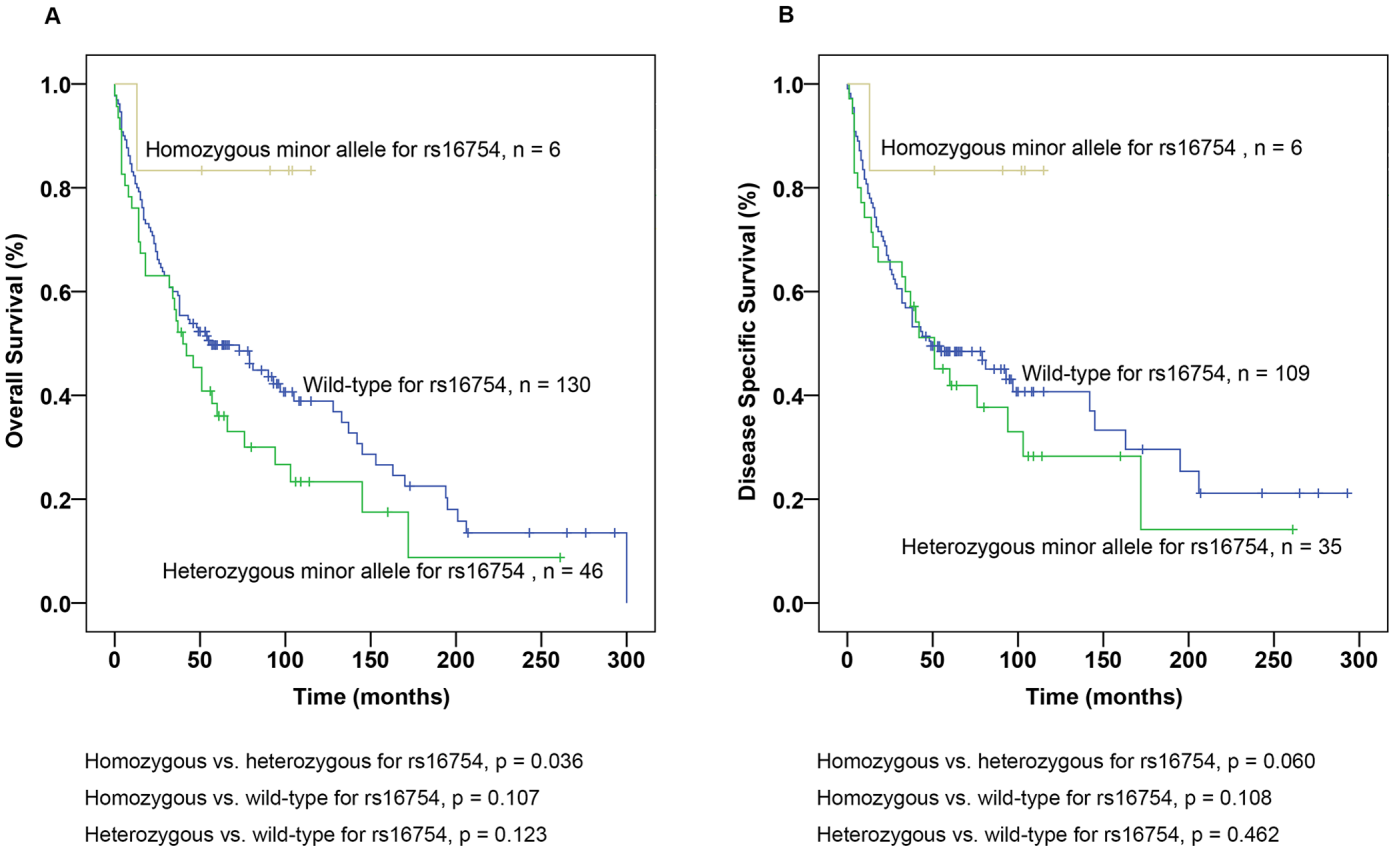
Prognostic impact of Wilms’ tumour gene 1 (*WT1*) single nucleotide polymorphism (SNP) rs16754 in ccRCC. (A) Overall Survival (OS) for patients with rs16754 wild-type, heterozygous and homozygous minor allele and (B) Disease-specific survival (DSS) for patients with rs16754 wild-type, heterozygous and homozygous minor allele.

With regard to *WT1* specific SNP genotype, *WT1* SNP rs2234581 was not evaluated due to too few cases (n = 2). There was no correlation between *WT1* genotype of the rs5030315 and outcome (data not shown). Subgroup analysis of the SNP rs16754 showed that patients with homozygous minor allele had significantly favourable OS compared to heterozygous genotype (p = 0.036, Figure 3A) and trend to longer DSS (p = 0.060, Figure 3B). In comparison with wild-type genotype, survival time did not differ significantly for patients with homozygous minor allele (p = 0.107 for OS and p = 0.108 for DSS, Figure 3A and B) and heterozygous genotype (p = 0.123 for OS and p = 0.462 for DSS, Figure 3A and B).

### 5. A Novel Missense Mutation

**Figure 4:**
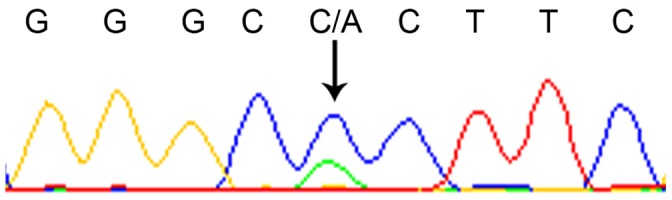
Chromatogram showing the nucleotide sequence with the novel heterozygous missense mutation 536 C→A in *WT1*.

Sequence analysis on 10 *WT1* exons did not show any insertions or deletions. Previous reported missense, nonsense or frame-shift mutations in *WT1* hot spots in leukaemia [19] or in Wilms’ tumour [31] were not identified in the present study. However, a novel heterozygous missense mutation in exon 1 at nucleotide position 536 C→A was identified in only one patient. This changes the amino acid from proline to histidine at codon 179 (Figure 4). *WT1* RNA expression was detected at a level of 0.048 (*WT1*/*β-actin* ×10^−3^). The patient was diagnosed with ccRCC at tumour stage IV and died shortly after diagnosis. The result showed that the common types of *WT1* mutations found in other types of malignancies were not demonstrated in ccRCC.

## Discussion

The prognostic significance of *WT1* mutation and *WT1* SNPs has been demonstrated in other types of malignancies, mostly in AML [19], [21]–[24], [32]. The present study evaluated *WT1* gene variations in ccRCC patients. We identified six different SNP genotypes, with 61% of patients harbouring at least one minor allele. *WT1* mutations commonly found in AML and Wilms’ tumour were not detected in our cohort of ccRCC patients. No association between SNP genotypes and *WT1* RNA expression level was found. We observed that patients with homozygous minor allele had favourable outcome compared to patients with wild-type and heterozygous minor allele. Similarly, longer OS and DSS were shown for patients homozygous for the minor allele for rs16754 compared to patients with heterozygous genotype. These results indicate that the prognostic impact of *WT1* SNPs in ccRCC is considerably weak due to too few patients harbouring the homozygous minor allele.

The International HapMap 3 consortium database has collected genetic data from the global population since 2002 (http://hapmap.nbci.nlm.nih.gov/) [33]–[34]. The minor allele frequency of *WT1* SNP rs2234582 was reported in 15.9% of HapMap European subjects. The minor allele frequency of rs2234582 in the present study was at a similar degree, with 16.8% of ccRCC tumour specimens and 16.8% of tumour-free specimens. In Asian populations, the minor allele frequency of rs2234582 was only 6.3% while the frequency in American populations is similar to that of Europe. SNP rs1799925 minor allele frequency was 15.3% in European subjects, 29.3% in American subjects and 69.6% in Asian subjects in the HapMap database. Similar to European subjects, 13.7% of the ccRCCs carried at least one copy of the minor allele for rs1799925 in the present study. The minor allele frequency of rs2234583 was reported in 5.9% of European subjects while we found the frequency to be about 6.6% in our tested samples. In Asian subjects, the frequency was only 1.2%. Similar minor allele frequencies of rs5030315 were detected in both our subjects and European subjects, while it was lower in Asian populations. In the present study, the rare SNP rs2234581 was indeed a rare finding, demonstrated in only two tumour samples. No collected data on rs2234581 could be found in the HapMap database. The minor allele frequency of rs16754 in the present study was 16% and demonstrated to be in agreement with data from the HapMap database for European subjects (15.9%). However, a Korean study found a significant difference in frequency compared to the German study [25]. The collected data suggest that the variation in rs16754 frequencies can be attributed to ethnicity of the group.

We have previously demonstrated that the *WT1* RNA expression was significantly lower in ccRCC compared to tumour-free renal cortical tissue, indicating that down-regulation of *WT1* acts as a tumour suppressor in ccRCC [10]. These findings were supported by a larger cohort in the present study. When relating *WT1* RNA expression to SNP genotypes, we did not find any correlation. It has been proposed that synonymous SNPs may change protein amount, structure or function through altering mRNA structure and stability, kinetics of translation and alternate splicing [35]. However, it is unclear whether RNA expression could be affected by the major or minor allele. One argument could be that an alternative *WT1* (*AWT1*) transcript, co-expressed with *WT1* in renal and hematopoietic cells [36], may affect detection of *WT1* using primers and probes in exon 1 and 2. In this study, primers and probes for measuring *WT1* RNA level were selected according to a quality-control study involving 11 European LeukemiaNet laboratories [37]. We tested multiple primers and probe sets spanning different *WT1* exons for 40 ccRCC samples and significant correlation was observed (data not shown). The mechanism of down-regulated WT1 expression in ccRCC may not be due to WT1 SNP genotypes. Other mechanisms, for example DNA methylation or related oncogenic pathways, may be involved in the decreased *WT1* expression in ccRCC. In contrast, *WT1* was found to be overexpressed in leukaemia [37]–[41]. Previous studies have suggested that *WT1* may play an oncogenic role in this type of tumour [42]–[43]. In AML patients, studies from Germany and the Netherlands found no significant differences in relation to *WT1* SNP rs16754 [23], [44], whereas a study by the Children’s Oncology Group presented that patients harbouring rs16754 minor allele had elevated *WT1* mRNA expression [32]. According to the Children’s Oncology Group, their findings may be explained by rs16754 genotype variations involving translation kinetics which could potentially affect protein folding and thereby protein function [32].

Studies have recently demonstrated that the minor allele of SNP rs16754 has a favourable effect on OS and relapse-free survival in AML [23]–[24], [32], [45]. This effect was most prominent for high-risk patients, defined as *FLT3-ITD* positive and/or *NPM1* wild type [23]. The authors hypothesized that the minor allele of rs16754 might be associated with increased drug sensitivity [23], thereby affecting patient outcome. In the case of the *MDR1* gene, a synonymous SNP altered the function of *MDR1* gene product P-glycoprotein despite similar mRNA and protein levels [46]. Similarly to the findings from AML studies, we observed that patients homozygous for the rs16754 minor allele had longer OS and DSS compared to patients heterozygous for the minor allele. Furthermore, patients carrying homozygous minor allele for SNPs in exon 1 also had favourable clinical outcome. Unfortunately, too few patients had the homozygous minor allele and therefore we were not able to evaluate whether the prognostic impact was independent in multivariable analysis. In rs16754, the rare codon CGA (6.2 per thousand) is replaced by the more frequently used codon CGG (11.4 per thousand, frequencies obtained from the Codon Usage Database [47]). Thus, the substitution of a rare codon for a more frequently used codon leads to increased translation kinetics that could potentially affect protein function as it has been demonstrated *in vitro* in *E. coli* [48]. In contrast with collected data on rs16754 as a positive prognostic factor, other studies demonstrated no significant impact in AML [25], [44].

In the present study, a novel missense mutation was identified in a patient with a stage IV ccRCC at diagnosis. In addition to the 536 C→A mutation, this patient also carried the heterozygous minor allele for the rs2234582 and rs16754. The *de novo* mutation was located in exon 1, coding for the proline/glutamine-rich transcriptional-regulation domain. This patient had a shorter survival time (4 months) than the median OS of 49.5 months in our study. This was the only *WT1* mutation detected in our subjects. Infrequent mutations of the *WT1* gene was reported by a Japanese study consisting of 34 primary urinary tract cancers including 22 RCC patients [49]. The results may imply that common *WT1* gene mutations are not involved in RCC.

In conclusion, our study has shown the occurrence of one or two copies of the *WT1* SNP minor allele in 61% of ccRCCs. In order of descending minor allele frequency, SNP rs2234582 was most frequent followed by rs16754, rs1799925, rs5030315, rs2234583 and rs2234581. No correlation was found between *WT1* SNP genotypes and clinical and pathological characteristics. However, differences in clinical outcome were observed between patients with homozygous minor allele versus heterozygous or wild-type. Considering its low frequency, larger confirmatory studies would be necessary.
